# Loosely-bound low-loss surface plasmons in hyperbolic metamaterial

**DOI:** 10.1186/s40580-018-0148-z

**Published:** 2018-06-08

**Authors:** Yu Shi, Hong Koo Kim

**Affiliations:** Department of Electrical and Computer Engineering and Petersen Institute of NanoScience and Engineering, University of Pittsburgh, Pittsburgh, PA 15261 USA

**Keywords:** Plasmonics, Surface plasmons, Metamaterials, Waveguides, Effective medium method

## Abstract

Surface plasmons (SPs) carry electromagnetic energy in the form of collective oscillation of electrons at metal surface and commonly demonstrate two important features: strong lateral confinement and short propagation lengths. In this work we have investigated the trade-off relationship existing between propagation length and lateral confinement of SP fields in a hyperbolic metamaterial system, and explored loosening of lateral confinement as a means of increasing propagation length. By performing finite-difference time-domain analysis of Ag/SiO_2_ thin-film stacked structure we demonstrate long range (~ 100 mm) propagation of SPs at 1.3 µm wavelength. In designing low-loss loosely-bound SPs, our approach is to maximally deplete electric fields (both tangential and normal components to the interface) inside metal layers and to support SP fields primarily in the dielectric layers part of metamaterial. Such highly-localized field distributions are attained in a hyperbolic metamaterial structure, whose dielectric tensor is designed to be highly anisotropic, that is, low-loss dielectric (Re(*ε*) > 0; Im(*ε*) ~ 0) along the transverse direction (i.e., normal to the interface) and metallic (large negative Re(*ε*)) along the longitudinal direction, and by closely matching external dielectric to the normal component of metamaterial’s dielectric tensor. Suppressing the tangential component of electric field is shown to naturally result in weakly-confined SPs with penetration depths in the range of 3–10 µm. An effective-medium approximation method is used in designing the metamaterial waveguide structure, and we have tested its validity in applying to a minimally structured core-layer case (i.e., composed of one or two metal layers). Low-loss loosely-bound SPs may find alternative applications in far-field evanescent-wave sensing and optics.

## Introduction

Supporting a surface-bound wave at metal/dielectric interface, plasmonic metals enable novel phenomena (e.g., negative refraction, field concentration and cloaking) [1–5]. Surface plasmons (SPs) commonly demonstrate relatively strong lateral confinement and short propagation lengths, for example, penetration depth of ~ 20 nm in metal and ~ 300 nm in dielectric side and propagation length of ~ 300 µm for the case of Ag/SiO_2_ interface at 1.3 µm wavelength. Whereas strong confinement of SP fields is viewed one of the most enabling nature of plasmonic phenomena widely exploited in near-field optics, short propagation lengths are a major limiting factor in exploring chip-scale (> ~ 1 cm) integration of plasmonic circuits and devices. The nature of this large plasmon loss is basically Ohmic, i.e., resistive, being caused by electron scatterings constantly occurring in metal [4–6]. The amount of energy loss, which eventually goes to Joule heating, can be expressed as $\omega {\text{Im}}\left( {\varepsilon_{m} } \right)\left| {E_{m} } \right|^{2}$, where *E*_*m*_ denotes electric field inside metal, ${\text{Im}}\left( {\varepsilon_{m} } \right)$ is the imaginary part of metal’s dielectric constant, and *ω* is angular frequency of light. In this work we have investigated the trade-off relationship existing between lateral confinement and propagation length of SPs supported in a hyperbolic metamaterial system and explored the opposite regime of SP phenomena, i.e., the case of loose confinement and long propagation length. In other words, loosening of lateral confinement is explored as a possible means of increasing propagation lengths for potential far-field optics applications.

In dire need of mitigating this intrinsic problem, i.e., large losses, plasmonics research community has exerted a great deal of efforts to extend propagation lengths into more practically useful ranges [7–25]: a variety of plasmonic waveguide structures have been proposed and demonstrated with improved performances, such as metal stripe, nanowires, V-grooves, gap, and dielectric/metal-layered structure. Among them, a thin-film metal/dielectric core waveguide structure, which is the subject of this current paper, is considered the most extensively studied: see, for example, Berini’s review paper on long-range surface plasmons and references therein [24]. In the early 1980s Sarid showed that a thin metal film sandwiched by symmetric dielectric cladding can support long-range SPs [7]: the SP fields supported by a thin metal core deeply penetrate into dielectric at both sides, and therefore the fraction of fields in the loss-inducing metal film part becomes insignificant, resulting in long propagation lengths. To support low-loss SPs the metal thickness needs to be typically smaller than penetration depth, ~ 20 nm. Due to the large ratio of dielectric constants of metal to dielectric, the normal *E*-field strength in metal is significantly weaker than that in dielectric, therefore, the confinement factor of beam power into the metal core is usually very small.

Stegeman and Burke [8] analyzed a double-electrode waveguide structure that comprises a dielectric layer sandwiched by two metal films forming a metal-dielectric-metal three-layer core structure. Four different types of surface-bound modes were identified, whose field distributions are governed by the types of symmetry involved in mutual coupling of SP fields bound to opposing metal surfaces. One of the symmetric modes (SC mode) shows excessively long propagation length (~ 10 mm) under the condition that the SP wave vector asymptotically approaches the propagation constant in external cladding dielectric and that the core dielectric thickness remains small. It is interesting to note that the authors identified the less-long-propagating mode (SS mode: ~ 1 mm) as the one carrying more technical importance. It is noteworthy that this double-metal-film core waveguide structure significantly enhances beam confinement into a core, when compared with the single thin-metal core structure case discussed above.

Recently Babicheva et al. [25] reported metal/dielectric multilayer-stacked hyperbolic metamaterial as a medium to support long-range SPs. By applying an effective medium approximation a metamaterial/dielectric interface is shown to support long-range plasmons when external dielectric becomes well-matched to the normal component of metamaterial’s dielectric tensor: $\varepsilon_{d} \cong {\text{Re}}\left( {\varepsilon_{m,n} } \right)$. A waveguide structure that comprises multilayer-stacked metamaterial as a core or cladding was also analyzed.

While a variety of metal/dielectric-stacked structures have been proposed for long-range surface plasmons, it is the current authors’ view that this subject field has been lacking a consistent approach to designing low-loss surface-plasmon waveguide structures. In this article we attempt to develop a simple unified understanding of how plasmon losses can be reduced/suppressed in metal/dielectric structures. It is noteworthy that the bulk of literature on long-range SPs have commonly reported observing a trade-off relationship between lateral confinement and propagation length, i.e., longer propagation lengths lead to more loosely-bound SPs. As a matter of fact this trade-off was recognized much earlier in an effort to design low-loss RF coaxial cables. In 1951, Clogston proposed to use a specially-designed metal/dielectric multilayer structure, a kind of metamaterial at RF frequency, in order to increase the field penetration into a metal core and thus to improve the signal propagation [26]. In the following year Black et al. [27] demonstrated this concept by developing a coaxial cable with a metal core surrounded by a multilayer metamaterial structure. However, in most literature in plasmonics field, which has been exploiting the strong confinement aspect of SP fields this trade-off has been viewed to be a drawback limiting the application potential to more conventional near-field optics, and has not been fully explored for alternative applications. In this paper we exploit this trade-off relationship and investigate the opposite regime of plasmon operation, i.e., loosely bound and low loss, as opposed to strongly bound and large loss in conventional SPs.

In establishing a design methodology applicable to a variety of different metal/dielectric layered structures, we are particularly interested in structures that involve a minimum number of metal layers. As an example of this minimal structure, we analyzed waveguide structures that employ a small number (one or two layers) of thin metal films (10-nm Ag) in the core layer part and demonstrate long-range (~ 100 mm propagation length) and loosely-bound (3–10 µm penetration depth) propagation of SPs. In designing the metamaterial waveguide structure we employ an effective-medium approximation method. Effective medium theory, in general, assumes a large number of periods of layered structure, and a natural question arises on its applicability to the case of metamaterial with a small number of periods. In this work we investigated the validity of this approximation applied to the double-layer metal core case. This result is then compared with that of alternative design of minimal structure, that is, a single metal layer core waveguide.

## Low-loss metamaterial structure

In designing low-loss plasmonic metamaterials that support loosely-bound SPs, our strategy is to suppress electric fields (therefore, Ohmic loss) in metals to a negligible level. Specifically we start with a metallodielectric hyperbolic metamaterial structure [28–33], and design the dielectric constants such that electric fields in metal layers become fully suppressed (*E*_*m*_ ~ 0) while desired surface-bound wave fields (primarily, normal fields) are maintained only in dielectric layers part of the metamaterial. This design requirement is met by exploiting an extra degree of freedom offered by an anisotropic metamaterial system, that is, by designing the dielectric tensor to be very different in two directions: low-loss dielectric along the transverse direction (normal to the surface); highly metallic in the longitudinal direction (parallel to the surface). The tangential (longitudinal) component of electric fields in metamaterial can be reduced to a negligible level by closely matching external dielectric to the transverse dielectric tensor, while the normal (transverse) component of electric field in metal layers is suppressed by employing a thin-film metal/dielectric stack possessing a large dielectric-constant ratio.

**Fig. 1:**
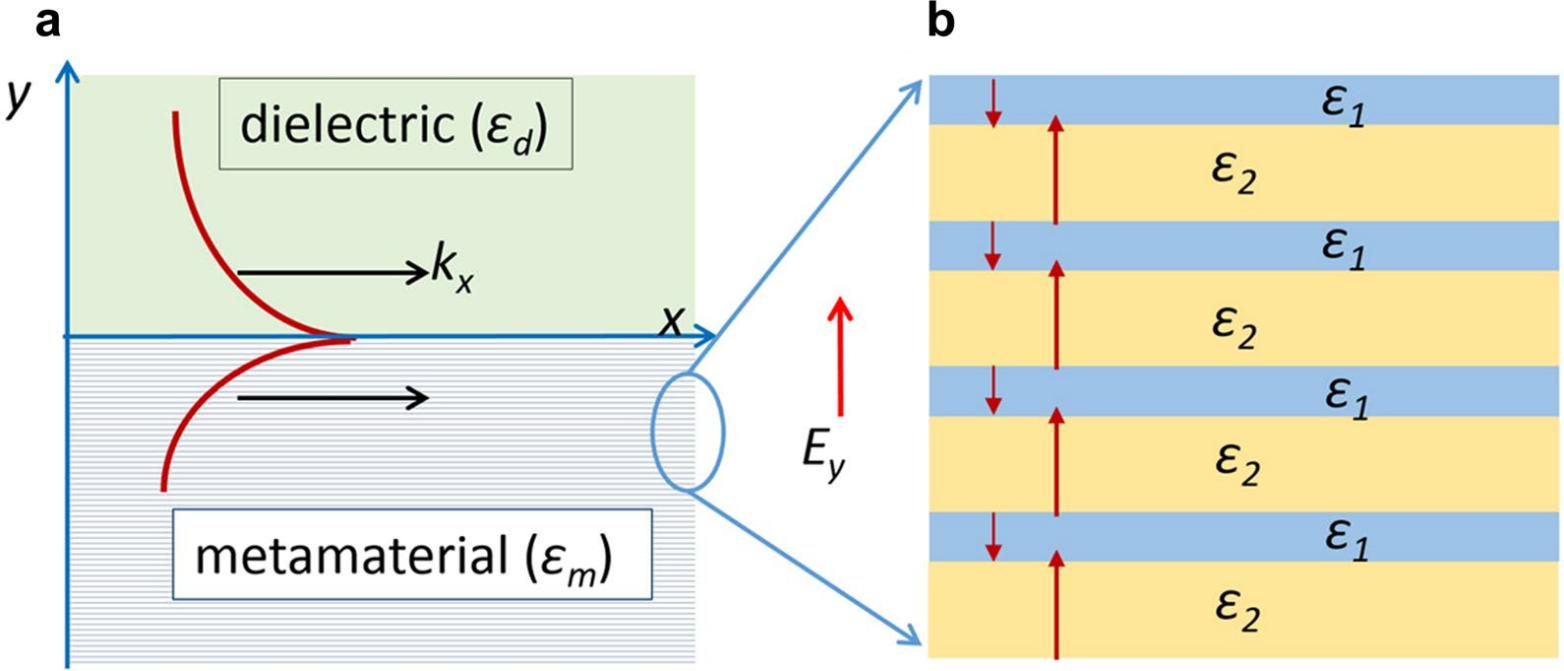
Surface-bound wave propagation at an interface of hyperbolic metamaterial (*ε*_*m*_) and isotropic dielectric (*ε*_*d*_). **a** Schematic of surface-plasmon field distribution. **b** Hyperbolic metamaterial composed of metal (*ε*_1_) and dielectric (*ε*_2_) thin-film multilayer stacked structure

Let’s imagine a surface-bound wave propagating along the interface of isotropic dielectric (*ε*_*d*_) and anisotropic uniaxial metamaterial (*ε*_*m*_), whose optical axis is aligned normal to the interface (Fig. 1): referring to Cartesian coordinates, the dielectric tensors take the following form, *ε*_*m,yy*_ = *ε*_*m,n*_ and *ε*_*m,xx*_ = *ε*_*m,zz*_ = *ε*_*m,t*_ for metamaterial, and *ε*_*d,xx*_ = *ε*_*d,yy*_ = *ε*_*d,zz*_ = *ε*_*d*_ for external dielectric. In view of the transverse nature (i.e., TM polarized) of surface-bound wave and referring to a wave vector expression $k = k_{t} \hat{t} \pm i\gamma_{n} \hat{n}$, the Maxwell’s equation ($\nabla \times H = \frac{\partial D}{\partial t}$) can be decomposed into two parts:

1 $$ k_{t} H = \omega \varepsilon_{n} E_{n} $$

2 $$ i\gamma_{n} H = { \mp }\omega \varepsilon_{t} E_{t} $$

where subscripts *n* and *t* denote the normal and tangential components, respectively, of fields (*E* and *H*), wave vector (*k*) and dielectric tensor (*ε*). From this equation set the surface-bound wave is predicted to possess the following properties: propagation characteristic (*k*_*t*_) is governed by normal component $(\varepsilon_{n} )$ of dielectric tensor, whereas transverse confinement (*γ*_*n*_) is determined by tangential component $(\varepsilon_{t} )$ of dielectric tensor. Applying a boundary condition to the interface it can be shown that the decay constant ratio of evanescent fields in both sides is determined by their dielectric constant ratio of tangential components:

3 $$ \frac{{\gamma_{d,n} }}{{\gamma_{m,n} }} = - \frac{{\varepsilon_{d} }}{{\varepsilon_{m,t} }} $$

In order to support low-loss loosely-bound SPs the metamaterial’s dielectric tensor is required to satisfy the following conditions: tangential component should be metallic (Re(*ɛ*_*m*,*t*_) < 0) for evanescent confinement in both sides (*γ*_*d*,*n*_, *γ*_*m*,*n*_ > 0); normal component should be low-loss dielectric (${\text{Re}}\left( {\varepsilon_{n} } \right) > 0;\;{\text{Im}}\left( {\varepsilon_{n} } \right) \sim 0)$ for long propagation lengths (${\text{Im}}\left( {k_{t} } \right) \sim 0)$; tangential *E*-field should be suppressed $\left( {E_{t} \sim 0} \right)$ for loose confinement $(\gamma_{n} \sim 0)$. These requirements can be met in a highly anisotropic hyperbolic metamaterial system.

The wave vector in each medium is governed by the following relationship:

4 $$ k_{t}^{2} /\varepsilon_{m,n} - \gamma_{m,n}^{2} /\varepsilon_{m,t} = k_{0}^{2} $$

5 $$ k_{t}^{2} /\varepsilon_{d} - \gamma_{d,n}^{2} /\varepsilon_{d} = k_{0}^{2} $$

where *k*_0_ is the free-space propagation constant. Combining these equations with the one derived above for a decay constant ratio we obtain the following expressions for propagation constant (*k*_*t*_) and decay constant (*γ*_*d,n*_ in dielectric side) of surface-bound wave:

6 $$ k_{t} /k_{0} = \sqrt {\varepsilon_{d} } \sqrt {(\varepsilon_{m,t} /\varepsilon_{d} - 1)/(\varepsilon_{m,t} /\varepsilon_{d} - \varepsilon_{d} /\varepsilon_{m,n} )} $$

7 $$ \gamma_{d,n} /k_{0} = \sqrt {\varepsilon_{d} } \sqrt {(\varepsilon_{d} /\varepsilon_{m,n} - 1)/(\varepsilon_{m,t} /\varepsilon_{d} - \varepsilon_{d} /\varepsilon_{m,n} )} $$

From Eq. (6) it can be shown that propagation constant *k*_*t*_ (= *k*_*x*_ = *β*) will asymptotically approach $\sqrt {\varepsilon_{d} } k_{0}$ as *ε*_*m,n*_ becomes equal to *ε*_*d*_. Presuming low-loss dielectric for *ε*_*d*_, the propagation constant *k*_*t*_ becomes positive real with a negligible imaginary part: ${\text{Re}}\left( {k_{t} } \right) > 0; \;{\text{Im}}\left( {k_{t} } \right)\sim 0$. This implies that we can achieve long propagation lengths (1/2Im(*k*_*t*_)) provided that normal component of dielectric tensor of metamaterial is closely matched to external dielectric ($\varepsilon_{m,n} \cong \varepsilon_{d}$). Similarly from Eq. (7), lateral decay constant $(\gamma_{n} )$ becomes zero (i.e., loosely confined) as *ɛ*_*d*_/*ɛ*_*m*,*n*_ approaches 1.

## Metallodielectric thin-film stack and effective medium approximation

Let’s consider implementing a uniaxial hyperbolic metamaterial system by stacking alternate layers of metal $\left( {\varepsilon_{1} } \right)$ and dielectric $\left( {\varepsilon_{2} } \right)$ thin-films in the vertical direction (Fig. 1, right). By applying an effective medium approximation the dielectric tensor (*ɛ*_*m*_) of the multilayered metamaterial can be expressed as follows:

8 $$ \varepsilon_{m,t} = f\varepsilon_{1} + (1 - f)\varepsilon_{2} $$

9 $$ \varepsilon_{m,n}^{ - 1} = f\varepsilon_{1}^{ - 1} + (1 - f)\varepsilon_{2}^{ - 1} $$

where *ɛ*_*m*,*t*_ denotes the dielectric constant along the in-plane tangential direction, and *ɛ*_*m*,*n*_ corresponds to the normal, thickness direction. $\varepsilon_{i}$ (*i* = 1, 2) represents the isotropic dielectric constant of component materials (*ɛ*_1_ for metal and *ɛ*_2_ for dielectric). *f* denotes the fraction of metal layer, that is, the ratio of metal thickness to bilayer period.

**Fig. 2:**
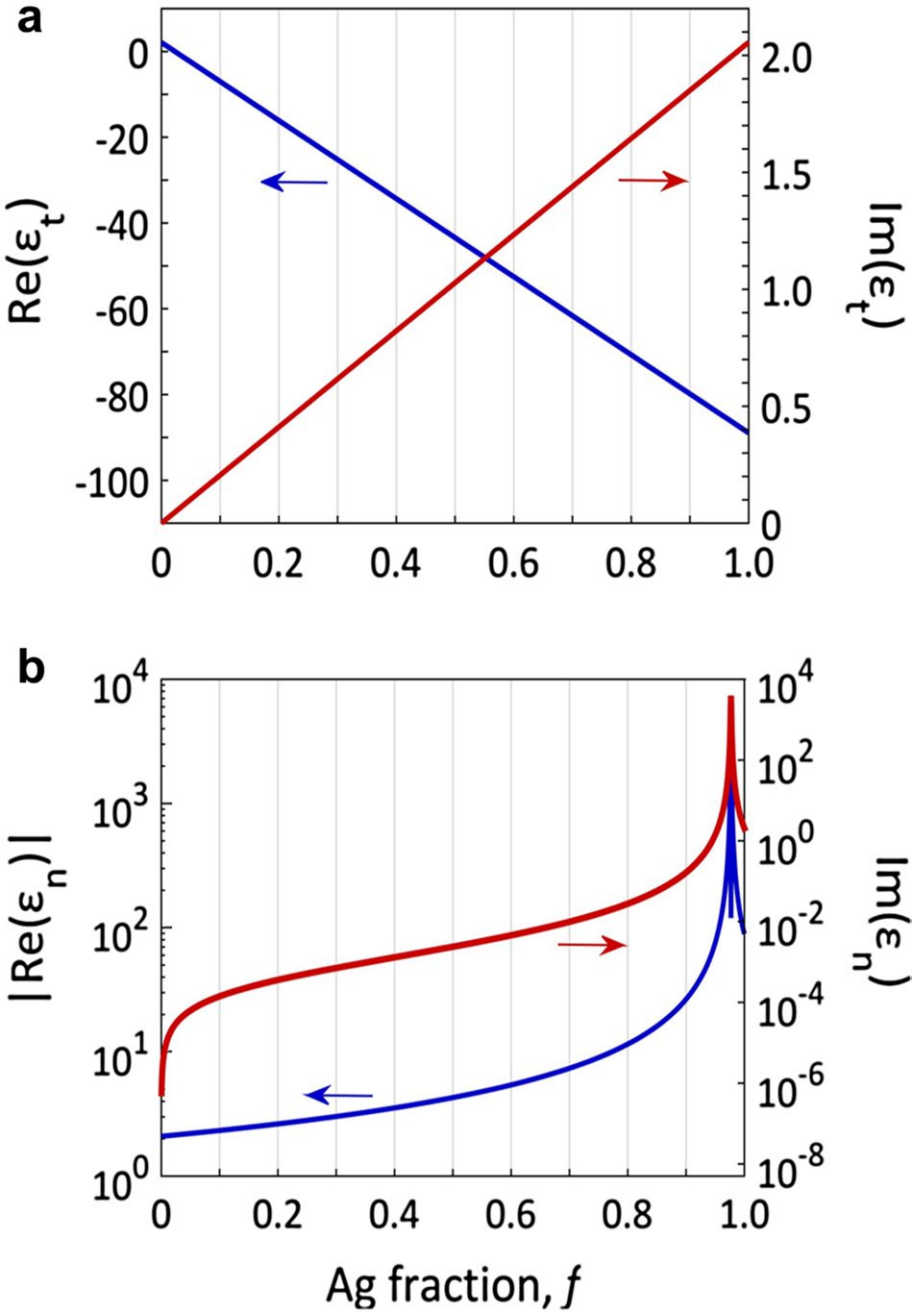
Dielectric tensors of Ag/SiO_2_-based hyperbolic metamaterial system calculated at 1.3 µm wavelength by applying an effective medium approximation. **a** Tangential component *ε*_*t*_: real part (blue; left axis) and imaginary part (red; right axis). **b** Normal component *ε*_*n*_: real part (blue; left axis) and imaginary part (red; right axis)

Figure 2 shows the normal and tangential components of dielectric tensors calculated for a Ag/SiO_2_ system. In this calculation the following dielectric constant values are assumed for constituent materials at 1.3 µm wavelength: *ɛ*_*Ag*_ = − 88.94 + *i*2.06; $\varepsilon_{{SiO_{2} }}$ = 2.09 [34, 35].

As a specific example, let’s consider the following composition: Ag-fraction, *f* = 0.1. The corresponding dielectric tensor is calculated to be: *ε*_*m,n*_ = 2.332 + *i*0.00014 and *ε*_*m,t*_ = − 7.01 + *i*0.2056. An optimum composition (metal fraction, *f*) of a given metamaterial system depends on external dielectric (*ε*_*d*_) that will be interfaced with the metamaterial: note that the Re(*ε*_*m,n*_) value of the chosen composition (*f* = 0.1) closely matches the dielectric constant of glass, e.g., soda-lime glass, *ε*_*d*_ = 2.28.

**Fig. 3:**
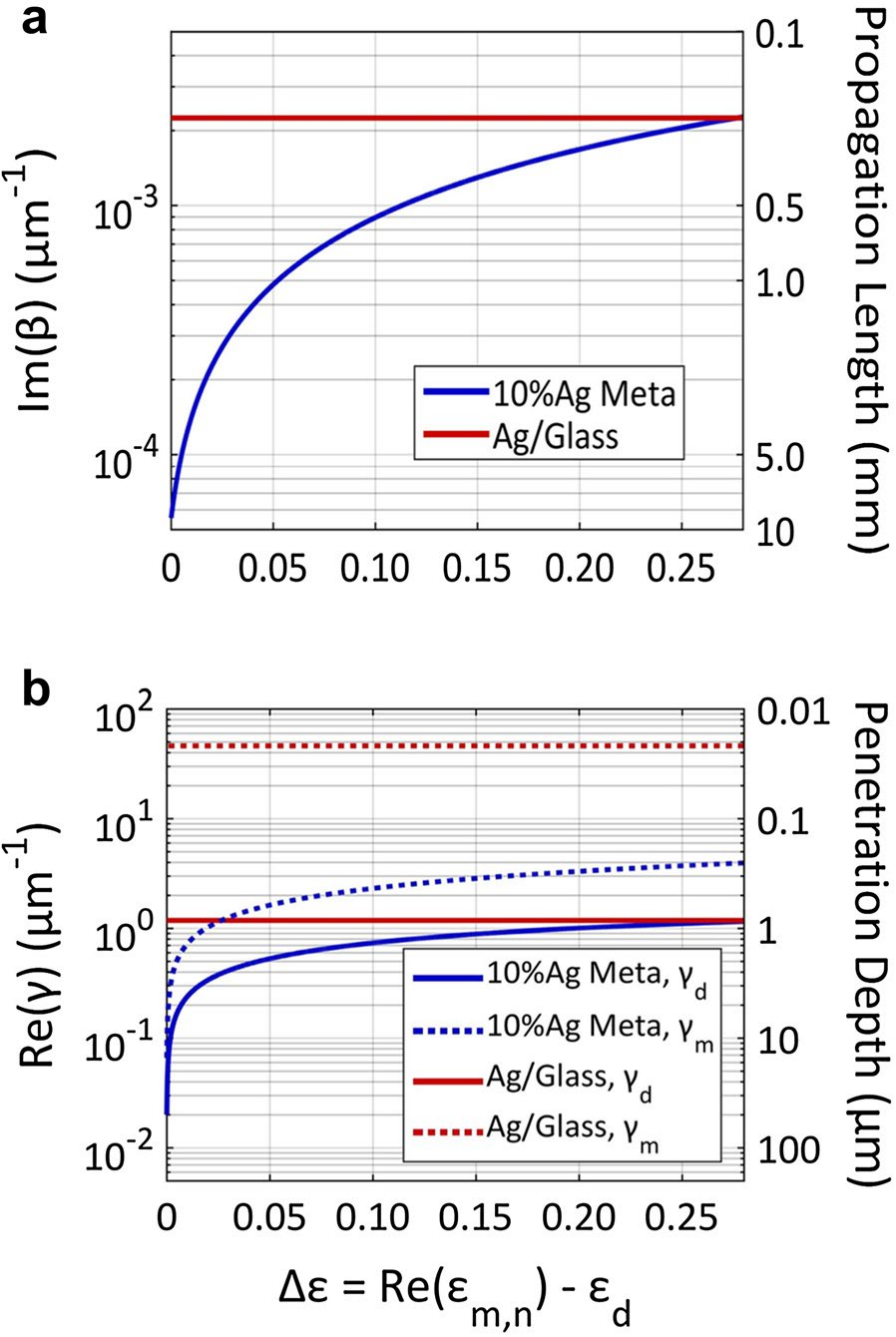
Surface-bound wave at an interface of hyperbolic metamaterial (*ε*_*m*_ with *f*_*Ag*_ = 0.1) and dielectric (*ε*_*d*_) calculated at 1.3 µm wavelength. **a** Propagation constant (*β*, left) and propagation length (1/2Im(*β*), right). **b** Lateral decay constant (*γ*, left) and penetration depth (1/Re(*γ*)), right) in dielectric side (solid) and metamaterial side (dotted). The case of bulk Ag on soda-lime glass (solid, red) is also shown for comparison

Now consider a surface-bound wave supported at an interface of Ag/SiO_2_-based metamaterial (*ε*_*m*_) and low-loss external dielectric (*ε*_*d*_). Figure 3 shows propagation constant (*k*_*t*_ ≡ *β*) and propagation length (1/2Im(*k*_*t*_)), and lateral decay constants (*γ*_*d*,*n*_, *γ*_*m*,*n*_) and penetration depths (1/Re(*γ*_*d*,*n*_), 1/Re(*γ*_*m*,*n*_)) of SPs calculated as a function of dielectric constant mismatch *nɛ* = *ɛ*_*m*,*n*_ − *ɛ*_*d*_. In this graph, external dielectric constant *ɛ*_*d*_ is varied while metamaterial dielectric tensor *ɛ*_*m*,*n*_ is fixed for a given composition *f*. When interfaced with a closely-matching dielectric (i.e., ∆*ɛ* = *ɛ*_*m*,*n*_ − *ɛ*_*d*_ ≅ 0) the metamaterial’s surface supports loosely-bound, long-propagating SPs as predicted above. In the case of *f*_*Ag*_ = 0.1, for example, SP propagation length is calculated to be 2.2 mm at ∆*ɛ* = 0.02 or 7.8 mm at ∆*ɛ* = 0.001 (blue in Fig. 3a), while penetration depth (into dielectric side) is estimated to be 2.9 µm at ∆*ɛ* = 0.02 or 13.1 µm at ∆*ɛ* = 0.001 (blue in Fig. 3b). These numbers correspond to 1.7 × 10^3^ to 6.0 × 10^3^ *λ* for propagation length and 2.3 to 10.0 *λ* for penetration depth (here, *λ* denotes free space wavelength).

It is important to note that a tradeoff relationship exists between propagation length and lateral confinement of surface-bound wave, that is, lateral confinement becomes weaker (i.e., more loosely bound) for longer propagation lengths. This relationship can be understood in view of the Maxwell’s equation discussed above, which relates transverse decay constant to tangential components of *E*-field and dielectric tensor: see Eq. (2). Lateral confinement becomes weaker (i.e., *γ*_*n*_ decreases) as tangential field (*E*_*t*_) is reduced, therefore, as propagation length increases.

This low-loss and loosely-bound behavior of SPs can be compared with those of conventional SPs as follows. Consider, for example, an interface of bulk Ag and glass at the same wavelength. Propagation length (1/2Im(*k*_*sp*_): $k_{sp} = k_{0} \sqrt {\frac{{\varepsilon_{m} \varepsilon_{d} }}{{\varepsilon_{m} + \varepsilon_{d} }}}$) is estimated to be 222 µm (red in Fig. 3a); penetration depth (into dielectric side, 1/Re(*γ*_*d*,*n*_)) is calculated to be 845 nm (red in Fig. 3b). This comparison shows that both propagation length and penetration depth can be simultaneously increased by orders of magnitude (> 10×) at properly-designed anisotropic metamaterial/dielectric interfaces.

## Wave-vector diagram for hyperbolic metamaterial

**Fig. 4:**
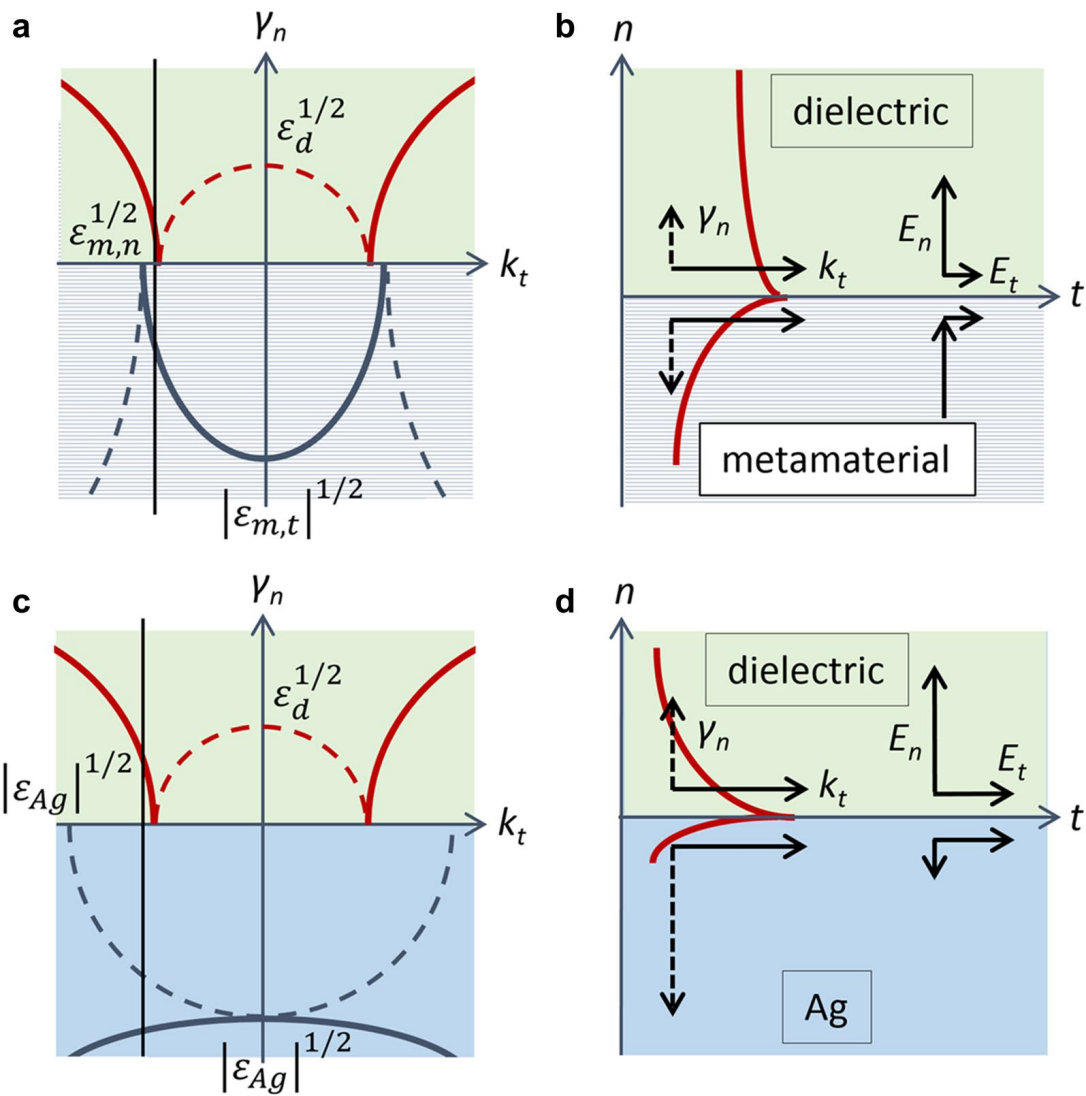
Wave-vector diagrams and surface-plasmon field distributions. **a** Phase-matching point (solid, black vertical line) for metamaterial/dielectric interface, and wave-vector diagram displayed in the (*k*_*t*_, *γ*_*n*_)-coordinate system. The red curve corresponds to wave-vector components (*k*_*t*_, *γ*_*n*_) in the dielectric side and the blue curve in the metamaterial side. The solid curve denotes evanescent waves and the dashed curve indicates propagating waves. **b** SP-field profile (*H*_*z*_, red), and the relative amplitude and orientation of normal and tangential component of *E*-field (right). Note the loose confinement of SP field and the dominance of normal *E*-field. **c** Phase-matching point (black vertical line) at bulk metal/dielectric interface. **d** SP-field profile (*H*_*z*_, red) and the relative amplitude and orientation of normal and tangential components of *E*-field (right). Note the strong confinement of SP field and the dominance of tangential *E*-field in metal side

The physical nature of the surface-bound waves supported by this hyperbolic metamaterial system differs significantly from the SPs at conventional bulk-metal surface, and this difference can be better understood referring to a wave-vector/phase-matching diagram (Fig. 4). This diagram basically depicts the following relationship of wave vectors ($k = k_{t} \hat{t} + k_{n} \hat{n} = k_{t} \hat{t} \pm i\gamma_{n} \hat{n}$), referring to *k*_*t*_ and *γ*_*n*_ in both sides of interface: $k_{t}^{2} /\varepsilon_{m,n} - \gamma_{m,n}^{2} /\varepsilon_{m,t} = k_{0}^{2}$ for hyperbolic metamaterial; $k_{t}^{2} /\varepsilon_{d} - \gamma_{d,n}^{2} /\varepsilon_{d} = k_{0}^{2}$ for isotropic dielectric. Here *k*_*n*_ denotes the normal component of wave vector *k*, while *γ*_*n*_ is the decay constant in the normal direction. Their relationship is given by *k*_*n*_ = ± *iγ*_*n*_: + for dielectric side and − for metamaterial (or metal) side in Fig. 4. Here it should be noted that both formula refer to the decay constants in the normal direction ($\gamma_{m,n} , \gamma_{d,n} )$, not the normal components of wave vector (*k*_*m*,*n*_, *k*_*d*,*n*_). Also the imaginary parts of *γ*_*n*_ and *k*_*t*_ are assumed to be negligible in this diagram. Solid curves (blue or red) indicate an evanescent field regime (i.e., *γ*_*n*_ remains positive real) and dashed curves correspond to a propagating/radiation mode regime (i.e., *γ*_*n*_ remains imaginary).

In order to support a surface-bound wave, that is, *γ*_*n*_ be positive real in both metamaterial and dielectric sides, the following condition should be met: *ɛ*_*d*_ < *ɛ*_*m*,*n*_ (Fig. 4a). To maintain long propagation lengths, the amount of dielectric mismatch (∆*ɛ* = *ɛ*_*m*,*n*_ − *ɛ*_*d*_) should be kept as small as possible. This indicates that the range of *k*_*t*_ value to support low-loss SPs is very narrow, and the corresponding *γ*_*n*_ values would be small in both sides. Applying a boundary condition (Eq. 3) to this diagram, a solution point (*k*_*t*_, *γ*_*n*_) can be specified in a narrow window marked with a vertical solid line: see Fig. 4a for the section where solid curves are in both sides, that is, surface bound.

Figure 4c shows a wave vector diagram of bulk metal/dielectric interface. Note that the dispersion curve in metal side is elliptical (circular), contrastingly different from the hyperbolic profile in metamaterial case. The metal side supports only evanescent wave (bottom side; blue, solid curve). The solution point can be found by applying the condition $\frac{{\gamma_{d,n} }}{{\gamma_{Ag} }} = - \frac{{\varepsilon_{d} }}{{\varepsilon_{Ag} }}$, and is marked with a vertical solid line. Because of the relatively large dielectric constant ratio of Ag/SiO_2_, the decay constant in Ag is very large, implying strong confinement of SP fields in metal side. According to Eq. (2), strong confinement (i.e., large *γ*_*n*_) implies presence of strong tangential *E*-field (i.e., large $E_{t}$). The ratio of tangential component to normal component of *E*-field is determined as:

10 $$ \frac{{E_{t} }}{{E_{n} }} = \frac{{ - i\gamma_{n} }}{{k_{t} }}. $$

For Ag/SiO_2_ at 1.3 µm wavelength, this ratio (|*E*_*t*_/*E*_*n*_|) is estimated to be 6.3 in metal side and 0.16 in dielectric side. By contrast, in the case of metamaterial/dielectric interface with *f* = 0.1 and ∆*ɛ* = 0.02, this ratio is estimated to be 0.046 in metamaterial side and 0.046 in dielectric side. This analysis indicates that the *E*-field in Ag is predominantly tangential (Fig. 4d, right), whereas normal *E*-field is dominant in well-matched metamaterial side (Fig. 4b, right). Overall this analysis indicates that the SPs in well-matched metamaterial/dielectric interface becomes quasi-transverse-electromagnetic (TEM), different from transverse magnetic (TM) of conventional SPs [11].

The nature of plasmon loss is Ohmic resulting in Joule heating, and the amount can be expressed as follows [36]:

11 $$ {\text{Re}}(J^{*} E) = \omega {\text{Im}}\left( {\varepsilon_{m} } \right)\left| {E_{m} } \right|^{2} = \omega {\text{Im}}\left( {\varepsilon_{m,n} } \right)\left| {E_{m,n} } \right|^{2} + \omega {\text{Im}}\left( {\varepsilon_{m,t} } \right)\left| {E_{m,t} } \right|^{2} . $$

In metal side (conventional bulk metal), both Im(*ɛ*_*m*,*n*_) and Im(*ɛ*_*m*,*t*_) take an equal, large number, and the presence of strong *E*-field (mostly tangential) in metal would result in large plasmon losses, mainly contributed by the 2nd term of Eq. (11). By contrast, in the case of well-matched metamaterial, both terms can be kept small: the low-loss dielectric constant of metamaterial in normal direction (i.e., ${\text{Im}}\left( {\varepsilon_{m,n} } \right) \sim 0$) suppresses the first term, while the second term is reduced with suppressed tangential *E*-field (i.e., $\left| {E_{m,t} } \right|\sim 0$). Overall this analysis confirms the importance of depleting electric fields (especially the tangential component) in metal layers in reducing plasmon losses.

**Fig. 5:**
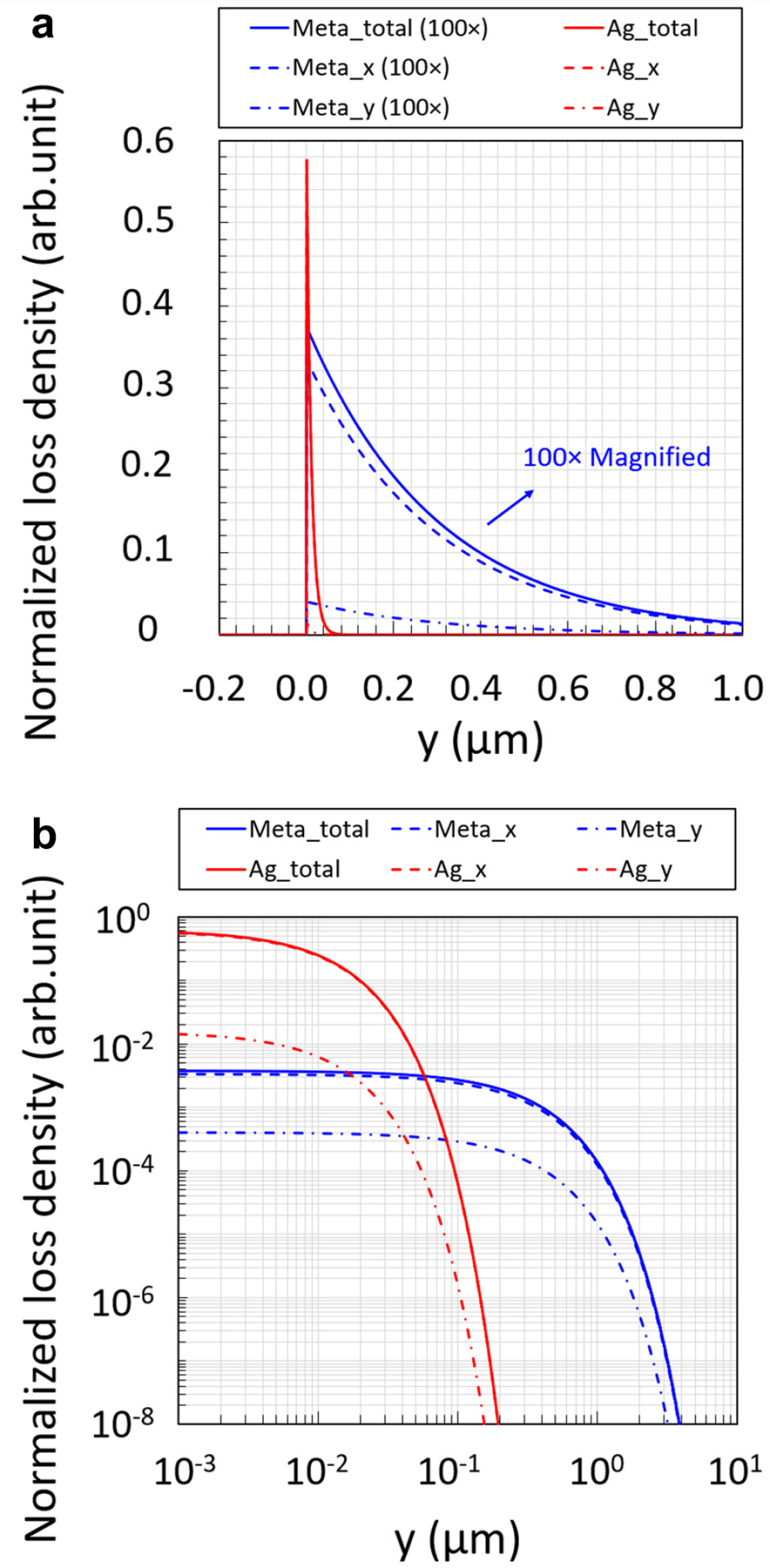
Normalized Ohmic loss power densities plotted as a function of distance (*y*) from interface. **a** Linear-scale plots. Glass side is y < 0. (red) Ag/glass. (blue) Metamaterial (*f* = 0.1)/glass. (solid) Total loss density. (dashed) Contribution by tangential electric field. (dash-dot) Contribution by normal electric field. Note that metamaterial losses are 100× magnified. **b** Log–log scale plots

Figure 5 shows normalized Ohmic-loss power densities calculated as a function of vertical distance *y* from the interface: the red curve for Ag/glass and the blue curve for metamaterial (*f* = 0.1)/glass case. In this calculation the Joule heating formula (Eq. 11) is normalized by the total energy flux stored in electric field ($\int_{ - \;\infty }^{ + \;\infty } {\omega \text{Re} (\varepsilon )\left| E \right|^{2} } dy$. Figure 5a is linear-scale plots revealing contrasting distributions of Ohmic losses in the metal and metamaterial side: an intense but narrow distribution in Ag; a weaker but wider distribution in metamaterial. Note also the negligible level of Ohmic loss in the dielectric side (*y* < 0). Figure 5b is log–log scale plots for more quantitative comparison. In the Ag case (red curve) the Ohmic loss is found to be dominated by the contribution from tangential *E*-field and the normal *E*-field contribution (dash-dot curve) remains negligible. In the metamaterial case the loss is still dominated by the tangential *E*-field component, mainly due to the large Im(*ɛ*_*m*,*x*_), but both field contributions are significantly lower than the Ag case. A normalized Ohmic loss is then calculated by integrating the loss-power density distributions along the depth direction (*y*) for Ag and metamaterial cases. Their ratio (Ag over metamaterial) is calculated to be 4.5. This number well matches the inverse ratio (4.51) of corresponding propagation lengths at the given materials interfaces: 223 μm for Ag versus 1002 μm for metamaterial (Fig. 3a: read the red curve at *Δε* = 0.052 for soda-lime glass).

## Field distributions in multilayer-stacked hyberbolic metamaterial

In order to elucidate the loosely-bound and low-loss nature of SPs we further analyzed the field distributions in hyperbolic metamaterial by performing finite-difference time-domain (FDTD) analysis on multilayer-stacked structures. On another aspect, this simulation study is also intended to test and validate the accuracy of the effective medium approximation applied to the multilayer structures discussed above. It should be noted that the effective medium approximation formula (Eqs. 8 and 9) assumes a constant field profile in each constituent layer. Considering the tendency to form evanescent profiles at metal/dielectric interface, each metal layer thickness is usually designed to be significantly smaller than penetration depth (~ 20 nm) so that the fields inside metal would remain nearly constant across the film thickness [29–33]. In implementing the metamaterial with *f*_*Ag*_ = 0.1 composition, for example, we considered alternately stacking 10-nm Ag and 90-nm SiO_2_ films, which is then interfaced with external dielectric (soda-lime glass, *ε*_*d*_ = 2.28) on one side. This metamaterial/dielectric interface corresponds to dielectric mismatch ∆*ɛ* = 0.052 (or 2.3% mismatch), and supports SPs with 1.0-mm propagation length and 1.8- or 0.6-µm penetration depth into glass or metamaterial side, respectively: see Fig. 3 blue curves.

**Fig. 6:**
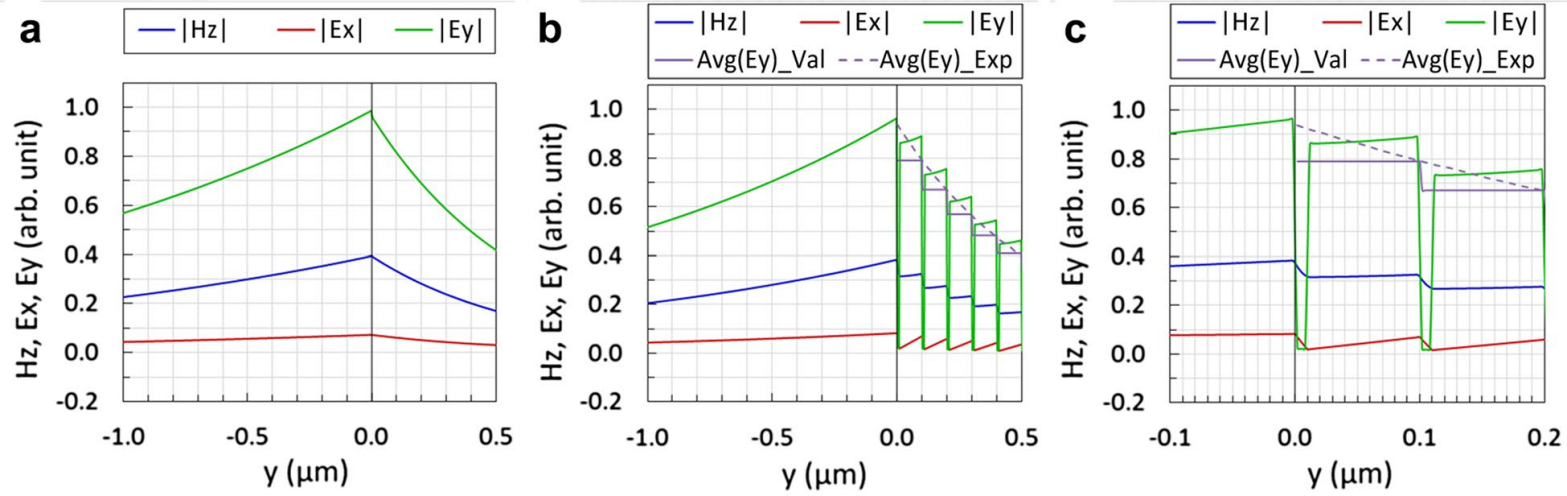
FDTD analysis of field distributions (*H*_*z*_, *E*_*y*_, and *E*_*x*_) in a hyperbolic metamaterial/dielectric structure. The metamaterial side (*y* > 0) consists of Ag(10 nm)/SiO_2_(90 nm) alternating multilayers. **a** A homogeneous anisotropic dielectric tensor, calculated by an effective medium approximation, is assumed for the metamaterial part. **b** Isotropic dielectric constants of bulk Ag and SiO_2_ are assumed for the multilayer structure. **c** A close-up view of panel (**b**) near the interface (*y* = 0). Note that normal *E*-field (*E*_*y*_: green) is well suppressed in metal layers while remaining strong in dielectric layers. Also note that tangential *E*-field (*E*_*x*_) remains low, implying that this surface-bound wave becomes more transverse-electromagnetic than transverse-magnetic

FDTD analysis was performed to calculate field distributions (*H*_*z*_, *E*_*y*_, and *E*_*x*_) of a metamaterial/dielectric structure at 1.3 µm wavelength by assuming two different dielectric tensors for the metamaterial part: (1) a homogeneous anisotropic dielectric tensor (*ɛ*_*m*_) calculated by applying an effective medium approximation; (2) isotropic dielectric constants of bulk materials (*ɛ*_*Ag*_, $\varepsilon_{{SiO_{2} }}$) for each component layers of the metamaterial. Figure 6 shows a comparison of the two simulation results calculated with: (a) *ɛ*_*m*_ for the metamaterial part; (b) *ɛ*_*Ag*_ and $\varepsilon_{{SiO_{2} }}$ for Ag and SiO_2_ layers, respectively; (c) a close-up view near the interface (*y* = 0) of multilayer simulation result [panel (b)]. In multilayer simulation case [panels (b and c)] the average field distributions inside metamaterial part (*y* > 0) are also shown for comparison (grey; solid for average value per period and dashed for exponential fitting).

First of all, both simulation results (homogenous versus multilayer) are in reasonable agreement, demonstrating similar penetration depths: 1.8 µm (homogeneous) versus 1.6 µm (multilayer) in glass, and 0.6 µm (homogeneous) versus 0.5 µm (multilayer) in metamaterial side. Use of smaller metal thickness (i.e., < 10 nm) for the metamaterial with the same metal composition (i.e., the same ratio of Ag thickness to bilayer period) would result in even better agreement. Here we iterate that a metal/dielectric-stacked structure naturally supports evanescent fields across each interface whereas the effective medium approximation formula (Eqs. 8 and 9) assume a flat distribution of fields in each constituent layer. This deviation of field profiles results in inaccuracy of effective medium theory. The total field distribution inside a given metal layer is basically a superposition of two evanescent fields stemming from both interfaces, and tends to be flat in the center region in the symmetric coupling case. As metal thickness is reduced below penetration depth (~ 20 nm in metal) this flattening effect becomes more significant, resulting in better accuracy of effective medium theory. In this study, however, we chose 10 nm as the minimum thickness, considering the technical difficulty of depositing continuous metal films at < 10 nm thickness. Overall this comparison validates the application of effective medium approximation to a metamaterial/dielectric system, provided that each Ag layer thickness (< ~ 10 nm) is designed to be much smaller than penetration depth and the dielectric constants (normal component) are well matched between metamaterial and external dielectric (e.g., ∆*ɛ* < ∼ 0.05). Tangential *E*-field (*E*_x_, red) remains globally low at an insignificant level; normal *E*-field (*E*_y_, green) is almost fully suppressed in metal layers, while maintaining its strength in dielectric layers (see Fig. 6c: zoom-in of b near interface). Note that field amplitudes (|*E*|, |*H*|) are plotted in this graph, and normal *E*-field (*E*_y_) in Ag layers orients to the opposite direction of that in SiO_2_ layers. Overall this field distribution analysis confirms that the electric fields in metal layers of properly-designed metamaterial can be depleted to a negligible level, resulting in low-loss propagation of loosely-bound SPs.

## Surface-bound waves in dielectric/metamaterial/dielectric waveguide structure

In designing three-layer (cladding/core/cladding) waveguide structures that support low-loss surface-bound waves at both interfaces of core layer, a hyperbolic metamaterial can be employed for either a core or cladding layer. From the implementation perspective, however, a metamaterial-core structure is preferred: this is because a metamaterial-cladding structure would require thicker metamaterial, therefore, more metal layers, although the metamaterial-cladding would, in general, allow stronger confinement of light in the lateral direction. In this work we focus on the metamaterial-core case with alternative application potential in mind, that is, to exploit the loosely-bound nature of low-loss surface plasmons. Further we are interested in the waveguide structures that will involve a minimum number of metal layers incurring lowest possible losses.

**Fig. 7:**
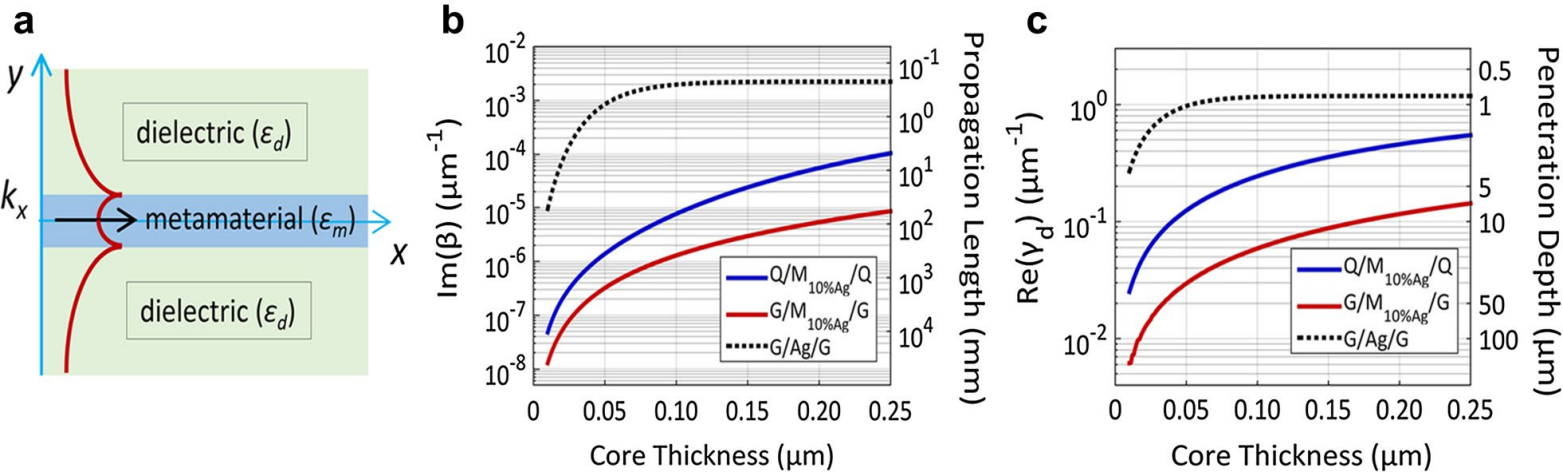
A three-layer (dielectric/metamaterial/dielectric) waveguide structure. **a** Schematic of surface-bound wave supported by a three-layer waveguide: Ag/SiO_2_-based hyperbolic metamaterial core (with *f*_*Ag*_ = 0.1) is sandwiched by silica (Q) or soda-lime glass (G) symmetric cladding. **b** Analytical calculation of propagation length for core thickness in the range of 10–250 nm. **c** Analytical calculation of penetration depth into cladding

Figure 7a shows a schematic of a three-layer waveguide structure that employs a metamaterial core sandwiched by dielectric cladding such as SiO_2_ (Q) or soda-lime glass (G). Here the waveguide core part is assumed to consist of Ag/SiO_2_/Ag three-layer thin-film stack with Ag composition *f*_Ag_ of 0.1, and is modeled as a homogeneous metamaterial possessing a dielectric tensor (*ε*_*m*_) that was calculated by applying an effective medium approximation at 1.3 µm wavelength: *ε*_*m,n*_ = 2.332 + *i*0.000141 and *ε*_*m,t*_ = − 7.01 + *i*0.2056. Note that this dielectric/metamaterial/dielectric structure corresponds to dielectric mismatch $\Delta \varepsilon$ of 0.238 or 0.052 for SiO_2_ or soda-lime glass cladding case, respectively. The following equations are solved to calculate propagation length and penetration depth into cladding of symmetric surface-bound mode supported by this three-layer waveguide structure.

12 $$ \frac{{\varepsilon_{d} }}{{\varepsilon_{m,t} }} + \frac{{\gamma_{d} }}{{\gamma_{m} }} \cdot tanh^{ - 1} \left( {\gamma_{m} \cdot {\raise0.7ex\hbox{$a$} \!\mathord{\left/ {\vphantom {a 2}}\right.\kern-0pt} \!\lower0.7ex\hbox{$2$}}} \right) = 0 $$

13 $$ \frac{{\beta^{2} }}{{\varepsilon_{m,n} }} - \frac{{\gamma_{m}^{2} }}{{\varepsilon_{m,t} }} = k_{0}^{2} $$

14 $$ \frac{{\beta^{2} }}{{\varepsilon_{d} }} - \frac{{\gamma_{d}^{2} }}{{\varepsilon_{d} }} = k_{0}^{2} $$

Here, *a* denotes the thickness of metamaterial core layer. Other parameters are the same as above. There can be multiple solutions of this transcendental equation set, but we will focus on the fundamental mode (symmetric and surface-bound), which demonstrates the lowest loss.

Figure 7b, c show the result of analytical calculation of propagation length and penetration depth for core thickness in the range of 10–250 nm. In the better-matched case (i.e., ∆*ɛ* of 0.052 for soda-lime glass cladding; red), 93-mm propagation length is attainable at 200-nm core thickness, while penetration depth into cladding is calculated to be 8.6 µm. In the case of silica cladding (∆*ɛ* of 0.238; blue) propagation length and penetration depth at the same core thickness (200 nm) are estimated to be 9.1 mm and 2.2 µm, respectively. Note that both propagation length and penetration depth monotonically increase as core thickness is reduced. The conventional bulk-metal core case (black dotted) is also shown for comparison: 223-µm propagation length and 845-nm penetration depth at 200-nm core (Ag) thickness.

**Fig. 8:**
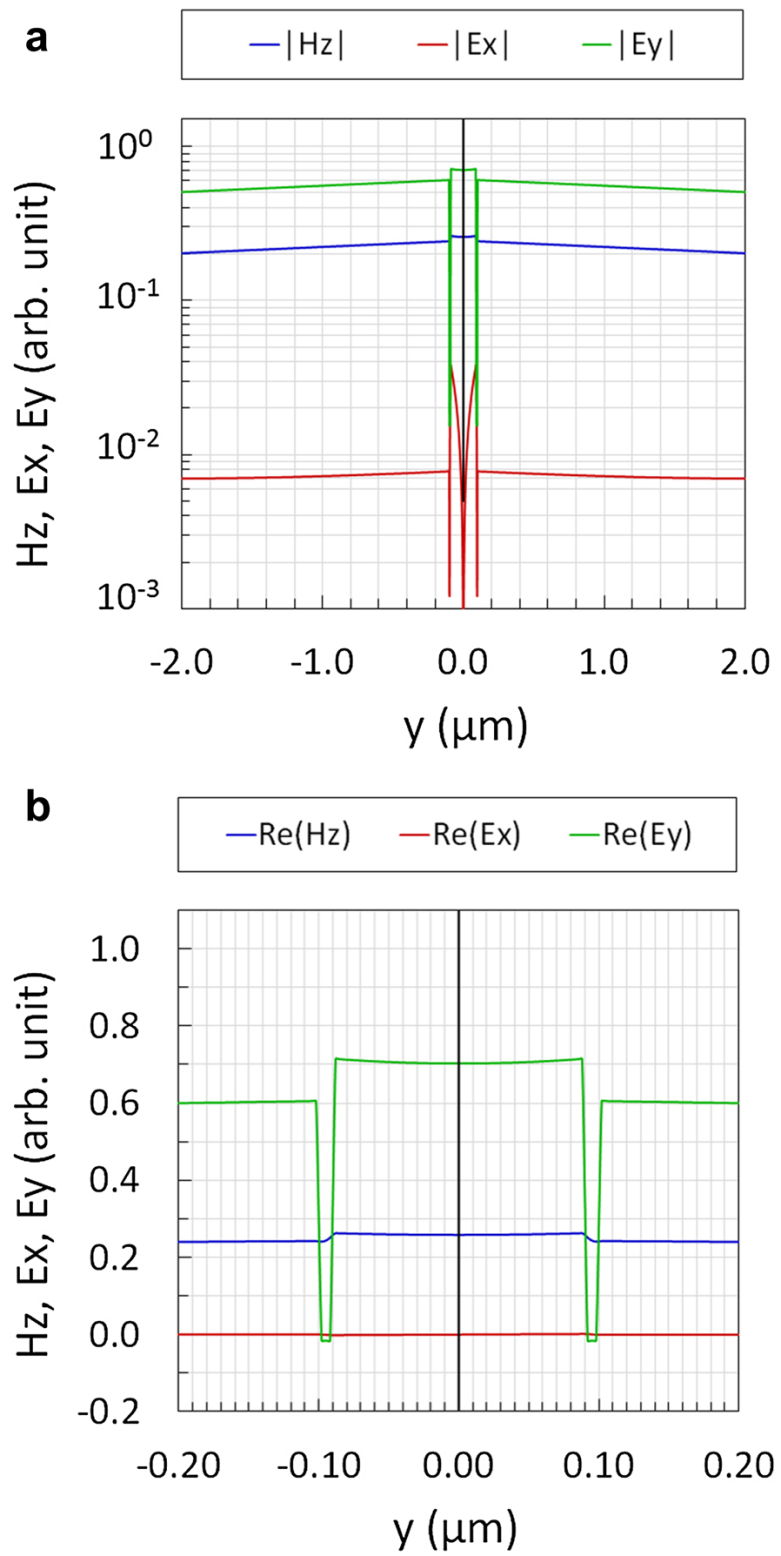
FDTD-calculated field distributions in a metamaterial-core waveguide structure: soda-lime glass/(10-nm Ag/180-nm SiO_2_/10-nm Ag)/soda-lime glass. **a** Field amplitude distributions of *H*_*z*_*, E*_*x*_ and *E*_*y*_ for y in the range of − 2 to 2 µm. Note the log scale of field amplitude. **b** A close-up view of core layer part (10-nm Ag/180-nm SiO_2_/10-nm Ag). Note that normal *E*-field (green) takes different signs in metal and dielectric layers

FDTD analysis was also performed on the original five-layer structure (glass/[Ag/SiO_2_/Ag]/glass) case, where the metamaterial core part (with *f*_Ag_ = 0.1) is assumed to consist of three layers (10-nm-Ag/180-nm-SiO_2_/10-nm-Ag) [8]. Figure 8 shows field distributions (*H*_*z*_, *E*_*y*_ and *E*_*x*_) calculated at 1.3 µm wavelength. From the *E*_*y*_ field plot (green in Fig. 8a) the field amplitude decays from 0.6 at *y* = 0.1 µm to 0.5 at *y* = 2.0 µm. Assuming an exponential decay profile the ratio of the two amplitudes can be expressed as: $\frac{{\left| {E_{y1} } \right|}}{{\left| {E_{y2} } \right|}} = \frac{{\left| {E_{y0} } \right|e^{{ - \frac{y1}{L}}} }}{{\left| {E_{y0} } \right|e^{{ - \frac{y2}{L}}} }}$. The penetration depth is then calculated to be:$L = \frac{y2 - y1}{{{ \ln }\frac{{\left| {E_{y1} } \right|}}{{\left| {E_{y2} } \right|}}}} = \frac{2 - 0.1}{{\ln \frac{0.6}{0.5}}} = 10.4\; \upmu {\text{m}}$. This number shows reasonable agreement with the analytical calculation (8.6 µm at 200-nm core thickness: see Fig. 7c). This comparison again validates the effective medium approximation applied to a well-matched metamaterial/dielectric system with a small number of constituent layers. Both tangential and normal components of *E*-field remain low in metal layers, whereas normal *E*-field maintains its strength in dielectric layers. The normal *E*-field profile (green) demonstrates a highly-localized (into dielectric layers) and yet broad (with large penetration depth) distribution, enabling low-loss propagation of SPs. Note also that normal *E*-field (*E*_*y*_) takes different signs in metal and dielectric layers: see Fig. 8b, green.

Next we analyzed the simplest (i.e., a single metal layer core) waveguide structure: a thin metal film core is sandwiched by symmetric dielectric cladding [7]. One might view this structure as a special case of the three-layer core waveguide structure discussed above: the thickness of spacer dielectric (SiO_2_) layer in the core part is reduced to zero under the assumption that the total (combined) metal thickness remains significantly smaller than skin depth (~ 20 nm). The dispersion characteristics in the core layer part, however, significantly differ between the two cases: elliptical for the single metal core case, whereas hyperbolic for the metamaterial core case. In terms of energy flow along the waveguide direction, the Poynting vector (time-averaged energy flow) in the core layer orients to the negative direction (backward) in the metal core case [15]. By contrast, in the metamaterial core case, the energy flow is in the positive direction (forward), the same as that in the cladding layers. The normal dielectric matching condition (∆*ɛ* ~ 0) is no longer applicable to this metal core case, and a symmetric surface-bound wave is always supported regardless of external dielectric constant. The governing equations of this three-layer waveguide structure with a thin-film metal core and symmetric dielectric cladding are given as follows:

15 $$ \frac{{\varepsilon_{d} }}{{\varepsilon_{m} }} + \frac{{\gamma_{d} }}{{\gamma_{m} }} \cdot tanh^{ - 1} \left( {\gamma_{m} \cdot {\raise0.7ex\hbox{$a$} \!\mathord{\left/ {\vphantom {a 2}}\right.\kern-0pt} \!\lower0.7ex\hbox{$2$}}} \right) = 0 $$

16 $$ \frac{{\beta^{2} }}{{\varepsilon_{m} }} - \frac{{\gamma_{m}^{2} }}{{\varepsilon_{m} }} = k_{0}^{2} $$

17 $$ \frac{{\beta^{2} }}{{\varepsilon_{d} }} - \frac{{\gamma_{d}^{2} }}{{\varepsilon_{d} }} = k_{0}^{2} $$

**Fig. 9:**
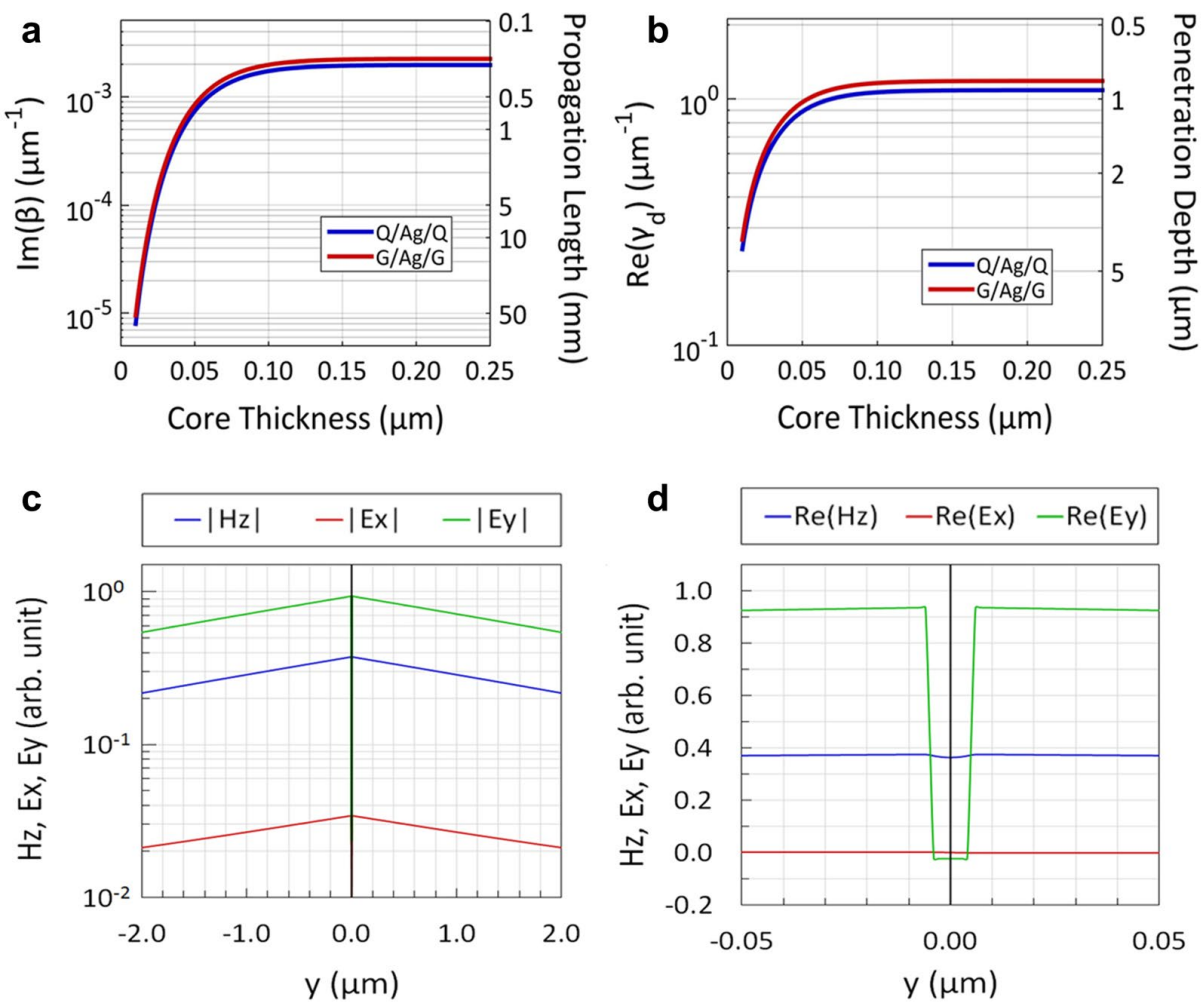
Analysis of a thin-metal-core waveguide structure with symmetric dielectric cladding: silica (Q) or soda-lime glass (G). **a** Analytical calculation of propagation length for metal core (Ag) thickness in the range of 10–250 nm. **b** Analytical calculation of penetration depth into cladding. **c** FDTD simulation of field distributions in SiO_2_/10-nm Ag/SiO_2_ structure: log-scale plot of field amplitudes of *H*_*z*_, *E*_*x*_ and *E*_*y*_ for y in the range of − 2 to 2 µm. **d** A close-up view of field distributions around the 10-nm Ag core

By solving the above equations we calculated propagation length and lateral confinement at 1.3 µm wavelength (Fig. 9a, b). In this calculation the Ag thickness was varied in the range of 10–250 nm and symmetric dielectric cladding is assumed to be silica (Q) or soda-lime glass (G). At 10-nm Ag thickness, propagation length of 52 mm or 63 mm is expected to be attainable for silica (Q) or soda-lime glass (G) cladding case, respectively, with corresponding penetration depth of 3.7 µm (Q) or 4.1 µm (G). As Ag film thickness is increased, both propagation length and penetration depth sharply decrease, asymptotically approaching conventional SPs’ at metal/dielectric interface.

Figure 9c, d show FDTD simulation result of *H*_*z*_, *E*_*y*_ and *E*_*x*_ field distributions for the case of 10-nm-Ag core with SiO_2_ cladding. Note that normal *E*-field *E*_*n*_ (= *E*_*y*_) is highly localized to dielectric layers. Also both *E*_*y*_ and *H*_*z*_ fields inside metal remain nearly flat across the metal thickness. This is because the evanescent profiles from both interfaces compensate their decaying profiles, resulting in a nearly constant profile across the metal thickness. As a result of this self-compensation effect, the tangential *E*-field (*E*_*t*_ = *E*_*x*_) becomes fully suppressed in most of the metal thickness. In other words, referring to the Maxwell’s equation, $i\gamma_{n} H_{z} = { \mp }\omega \varepsilon_{t} E_{t}$, the tangential *E*-field (*E*_*t*_) is reduced to zero as the magnetic field (*H*_*z*_) profile becomes flat ($\gamma_{n} \sim 0)$. Overall, suppressing the tangential *E*-field results in low-loss propagation of SPs, and this is enabled by employing a thin metal core with symmetric dielectric cladding. Unlike the metamaterial-core case, this thin-metal core structure does not require the condition of good dielectric-matching between core and cladding. Propagation length and penetration depth of a thin-metal core case are a strong function of metal thickness at < 50 nm range, but are less dependent on cladding dielectric constant (Fig. 9a, b). By contrast, in the metamaterial core case, the opposite characteristics (i.e., sensitive to dielectric matching and less sensitive to core layer thickness) are observed, and significantly longer propagation lengths and penetration depths are attainable: e.g., 100–200 mm propagation length and 10–15 µm penetration depth at 120–150 nm core layer thickness with ∆*ɛ* of 0.238 (see Fig. 7b, c). In terms of practicality of implementing the designed structure with good reproducibility (i.e., less prone to process fluctuation such as thickness variation), the metamaterial core structure with a small number of layers offers an advantage over the thin-metal core case.

## Conclusions

We have investigated the trade-off relationship existing between propagation length and lateral confinement of surface-bound waves in a hyperbolic metamaterial system, and explored loosening of lateral confinement as a means of increasing propagation length. By performing finite-difference time-domain (FDTD) analysis of Ag/SiO_2_ thin-film stacked structures we demonstrate long range (~ 100 mm) propagation of surface plasmons at 1.3 µm wavelength. In designing low-loss loosely-bound SPs, our approach is to maximally deplete electric fields (both tangential and normal components to the interface) inside metal layers and to support SP fields primarily in the dielectric layers part of metamaterial. Suppressing the tangential component of electric field naturally results in weakly-confined, quasi-TEM waves with penetration depths in the range of 3–10 µm. When designed into a stripe geometry of proper width, the loosened lateral confinement (i.e., penetration depth ~ 4 µm) across a metamaterial core would provide a good match to the modal size (~ 8 µm diameter) of silica-based single-mode optical fiber. Further the quasi-TEM mode supported by this waveguide structure maintains its polarization (with *E*-field oriented normal to metal film) over long-length propagation. Low-loss loosely-bound SPs may find alternative applications in far-field evanescent-wave sensing and optics as well.
